# Wound infection caused by *Staphylococcus arlettae*: a case report and metal characterization

**DOI:** 10.3389/fcimb.2026.1780396

**Published:** 2026-03-05

**Authors:** Tao Zhu, Yuanling Jin, Qiankun Liu, Jun Zhang, Qianglong Pan, Haixia Tu, Yan Li, Shouxing Wang

**Affiliations:** 1 Department of Clinical Laboratory, Sir Run Run Hospital, Nanjing Medical University, Nanjing, China; 2 Sir Run Run Hospital, Nanjing Medical University, Nanjing, China

**Keywords:** antibacterial resistance, biofilm, metal characterization, *Staphylococcus arlettae*, wound infection

## Abstract

**Background:**

Retained metallic foreign bodies can lead to implant-associated wound infections through bacterial colonization and biofilm formation. We report a case of a wound infection associated with a retained metallic fragment caused by *Staphylococcus arlettae* (*S. arlettae*) and evaluate the organism’s early biofilm formation on common implant metals.

**Case presentation:**

A 33-year-old man sustained a crush injury to his right hand and forearm, resulting in extensive soft-tissue damage and vascular injury. Emergency surgical management included meticulous debridement and vascular reconstruction. Postoperatively, purulent wound infection was effectively managed following microbiological identification of *S. arlettae* and antibiotic susceptibility-guided therapy. The treatment regimen involved serial debridement along with stepwise adjustments in antimicrobial dosing. Follow-up revealed that the patient’s hand function had recovered well.

**Methods and results:**

*In vitro* assays were conducted to compare early bacterial attachment and biofilm formation of the clinical *S. arlettae* isolate on stainless steel 304 (SS304), stainless steel 316 (SS316), and titanium alloy (TC4). The results revealed material-dependent differences in initial adherence as well as early biofilm development, establishing a link between implant surface properties and bacterial colonization propensity.

**Conclusions:**

This case underscores the clinical significance of retained metallic fragments as potential foci for *S. arlettae* infection, emphasizing the necessity for prompt debridement, targeted antimicrobial therapy, and consideration of implant material properties. *In vitro* evidence demonstrating differential biofilm behavior on SS304, SS316, and TC4 has important implications for surgical decision-making, selection of implants, management of wounds, and prophylactic antibiotic strategies aimed at mitigating implant-associated infections.

## Introduction

Penetrating and puncture wounds of the hand or wrist are common reasons for emergency department visits, making this anatomical region particularly susceptible to the retention of foreign materials following trauma. Previous studies have reported that 15–38% of foreign bodies are overlooked during initial clinical assessment (Anderson et al., 1982; Steele et al., 1998). Retained metallic fragments may serve as a surface for bacterial adhesion and subsequent biofilm formation, thereby increasing the risk of persistent or recurrent wound infection. In this context, infections caused by organisms such as coagulase-negative staphylococci, particularly the rare *Staphylococcus arlettae* (*S. arlettae*), may be underdiagnosed in clinical practice, as they are sometimes misinterpreted as contaminants rather than true pathogens.

*S. arlettae* was first isolated in 1984 from poultry and goats, and has subsequently been described in numerous reports (Schleifer et al., 1984). *S. arlettae* strains have been isolated from various animals (mainly mammals and birds), salt mines, estuaries, fermented foods, biosafety cabinets, and other environments (Tu et al., 2010; Chauhan and Garlapati, 2013; Adkins et al., 2018; Lavecchia et al., 2019; Pereira and Ramaiah, 2019). In recent years, *S. arlettae* strains have been isolated from different types of clinical specimens, including blood, intraoral sutures, cerebrospinal fluid, and swabs (Dinakaran et al., 2012; Teeraputon et al., 2017; Pandey and Chakraborty, 2021; Chukwumah et al., 2022; Kherdekar et al., 2023). To date, reported *S. arlettae* infections exhibit three key clinical characteristics: frequent association with foreign materials or implants, a tendency for deep involvement following invasive procedures, and widespread environmental distribution that increases exposure risk in both clinical and community settings.

This report aims to describe a case of *S. arlettae* wound infection associated with a retained metallic fragment and to investigate the bacterium’s adhesion and biofilm behavior on different metallic surfaces. It is hoped that the findings will contribute to an increased awareness of the issues associated with metal retention in wounds and the development of more effective clinical treatment strategies, thereby reducing the risk of future complications.

## Case presentation

### The case

A 33-year-old male patient with no history of familial genetic or infectious diseases was admitted to the hospital due to pain and bleeding in his right hand and forearm following trauma 20 minutes before. He reported that his right hand and forearm had been accidentally struck by a water pipe, resulting in immediate pain, bleeding, and restricted movement. After performing simple self-bandaging, he presented to the hospital, where he was admitted to the department with a preliminary diagnosis of “right hand injury.” The patient’s condition was considered fair during the admission process. He had no history of coma, reported good appetite and sleep, normal bowel and urinary habits, no chills or fever, no abdominal distension or pain, no cough or sputum, and no significant recent weight loss.

### Clinical data collection

Specialized physical examination revealed evident deformities and significant swelling of the right hand and forearm. A 7 cm wound on the ulnar palmar side of the right index finger and an 8 cm wound on the middle finger were visible, with each extending from the metacarpophalangeal joint to the fingertip. Both wounds were deep, down to the bone, and were heavily contaminated. There was a 4 cm transverse wound near the proximal ends of the third and fourth metacarpals, extending down to the tendons and heavily contaminated, as well a 4 cm wound and an oblique 3 cm wound were present on the proximal ulnar palmar side of the right wrist, extending to the bone and deep fascia layer, respectively, with numerous metal fragments visible. In addition, an irregular 5 cm wound on the proximal ulnar dorsal side of the right wrist, extending to the fracture site, was observed, with exposed tendons, visible metal fragments, and heavy contamination. Movement of the right fingers and wrist was restricted. Significant swelling was noted in the right hand and wrist palm area. Bone crepitus was palpable and audible. Obvious tenderness and pain on percussion were present, accompanied by severe skin contusion. Skin sensation was diminished in the distal segments of the right index and middle fingers. All fingers were ruddy in color, with normal capillary refill. No other significant abnormalities were noted. The Early Wound Healing Score (EHS) upon admission was Grade 2.Emergency X-ray findings indicated fractures of the right hand at the 2nd proximal and distal phalanges, 3rd-5th metacarpals, and 3rd-4th distal phalanges, as well as fractures of the right distal radius and ulnar styloid process. Laboratory results showed an elevated white blood cell count of 10.35×10^9^/L, with a neutrophil count of 7.64×10^9^/L, the red blood cell count was 4.98×10¹²/L, hemoglobin was 145 g/L, and the platelet count was 163×10^9^/L. Furthermore, the levels of D-dimer were abnormally elevated to 502 ng/mL, while those of fibrinogen were decreased to 2.04 g/L. Biochemical tests showed that creatine kinase (323 U/L), uric acid (457 µmol/L), and triglycerides (1.80 mmol/L) were elevated, while other indices were within normal ranges.

### Diagnostic evaluation and treatment

Upon admission, to prevent fracture-related infection, the patient received intravenous infusions of cefotiam 1 g/day and anisodamine hydrobromide 10 mg/day, along with intravenous injection of ketorolac tromethamine 30 mg/day for analgesia and to counteract inflammation. Subsequently, debridement of the right hand and forearm, along with exploration and repair of the tendons, blood vessels, and nerves, and internal fixation of fractures, were performed under brachial plexus block anesthesia. The surgical procedure was as follows: after successful anesthesia, the wounds were irrigated and trimmed. This was followed by thorough debridement and removal of foreign metal bodies. After re-irrigation, the ruptured tendons and nerves were repaired, fractures and dislocated joints were reduced, and fixation was performed using Kirschner wires, while closed reduction and cross K-wire fixation were applied to the distal radius. Intraoperative X-ray fluoroscopy confirmed satisfactory reduction and stable fixation of all fractures; the wounds were sutured with No. 0 silk thread, followed by sterile dressing and immobilization with a plaster cast.

### Microbiological identification

**Figure 1:**
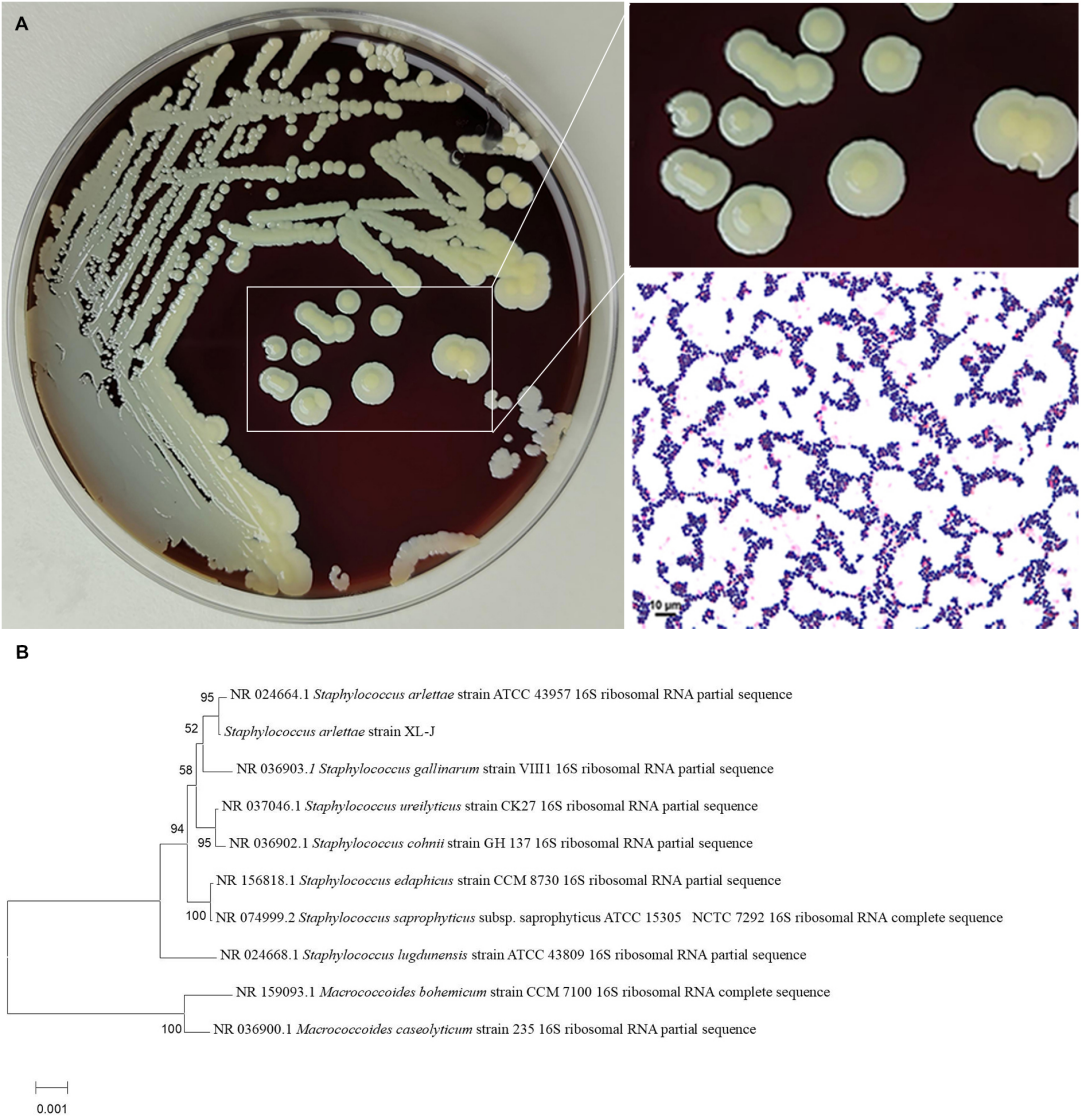
Observation of *S. arlettae* colonies on a Columbia blood agar plate and microscopic examination of a Gram-stained smear revealed Gram-positive cocci at 1,000 × magnification **(A)**. Neighbor-joining phylogenetic trees constructed by using 16S rRNA sequences of *S. arlettae* strains from patients in the hospital. *Macrococcoides caseolyticum* strain 235 and *Macrococcoides bohemicum* strain CCM 7100 represent the root of the tree **(B)**.

Sequential wound cultures were obtained from the proximal ulnar–volar aspect of the right wrist on hospital day 1 and postoperative day 5, both of which yielded *S. arlettae* only. Specifically, on hospital day 1, during wound debridement, a swab specimen was collected from deep soft tissue extending to the bone surface. On postoperative day 5, due to delayed wound healing, a repeat swab was obtained from the same deep wound site postoperatively. Culture of the wound swab specimens yielded abundant colony growth on Columbia blood agar plates. The colonies were opaque, yellowish-white, with entire edges, and measured 6–8 mm in diameter. Gram staining of a smear revealed Gram-positive cocci. The isolate was identified as *S. arlettae* with 99% confidence using the VITEK^®^ 2 Compact system (bioMérieux, France). Following 16S rRNA sequencing of the pure colonies by Sangon Bio Co. Ltd., the *S. arlettae* strain XL-J (GenBank: PX472910.1) was analyzed using NCBI BLAST, showing 99.86% similarity with *S. arlettae* strain SA283 (GenBank: CP097887.1) (**Figure 1**).

### Antimicrobial susceptibility testing

One week after the operation, the patient reported significant pain in the right hand wound, with a Visual Analog Scale (VAS) score of 5. The hand remained immobilized in the plaster cast and was dressed with medical chitosan and gauze combination dressings. However, copious light-yellow exudate was observed on the lateral aspect of the wound, accompanied by swelling. The minimum inhibitory concentrations (MICs) of antibiotics against the *S. arlettae* isolate were determined (**Table 1**). This indicated that the isolate was sensitive to chloromycetin, ciprofloxacin, daptomycin, gentamicin, levofloxacin, linezolid, moxifloxacin, furantoin, rifampicin, ticoranin, tetracycline, tigecycline, and vancomycin, but was resistant to ampicillin, cefoxitin, clindamycin, erythromycin, oxacillin, benzylpenicillin G, and cotrimoxazole.

**Table 1:** Antimicrobial susceptibility testing results.

Antimicrobial agent	*S. arlettae* strain XL-J	
	MIC(μg/ml)	Interpretation
Ampicillin	≤0.5	R
Cefoxitin	≤1	R
Chloromycetin	≤4	S
Ciprofloxacin	≤0.12	S
Clindamycin	1	R
Daptomycin	1	S
Erythromycin	>8	R
Gentamicin	≤2	S
Levofloxacin	≤1	S
Linezolid	2	S
Moxifloxacin	≤0.12	S
Furantoin	≤16	S
Oxacillin	2	R
Benzylpenicillin G	1	R
Rifampicin	0.5	S
Ticoranin	2	S
Tetracycline	≤1	S
Tigecycline	0.25	S
Cotrimoxazole	>4/76	R
Vancomycin	1	S

Susceptibility breakpoints for Staphylococcus isolates were determined using the criteria set forth in the Clinical and Laboratory Standards Institute (CLSI) M100 guidelines. S, susceptible; I, intermediate; R, resistant.

Considering the extensive wound area resulting from debridement and suturing, which posed a high risk for opportunistic infection during dressing changes, cefotiam, which is effective against Gram-positive cocci and some Gram-negative bacilli, was administered again post-operatively for an extended period. The dosage was increased from 1 g/day to 1 g/8 h. Supportive treatments included ketorolac tromethamine and rotundine sulfate for pain relief, nicotinic acid and mecobalamin for promoting blood circulation, mannitol for reducing edema, anisodamine hydrobromide for antispasmodic effects, and ossotide for promoting fracture healing.

### Follow-up and outcomes

**Figure 2:**
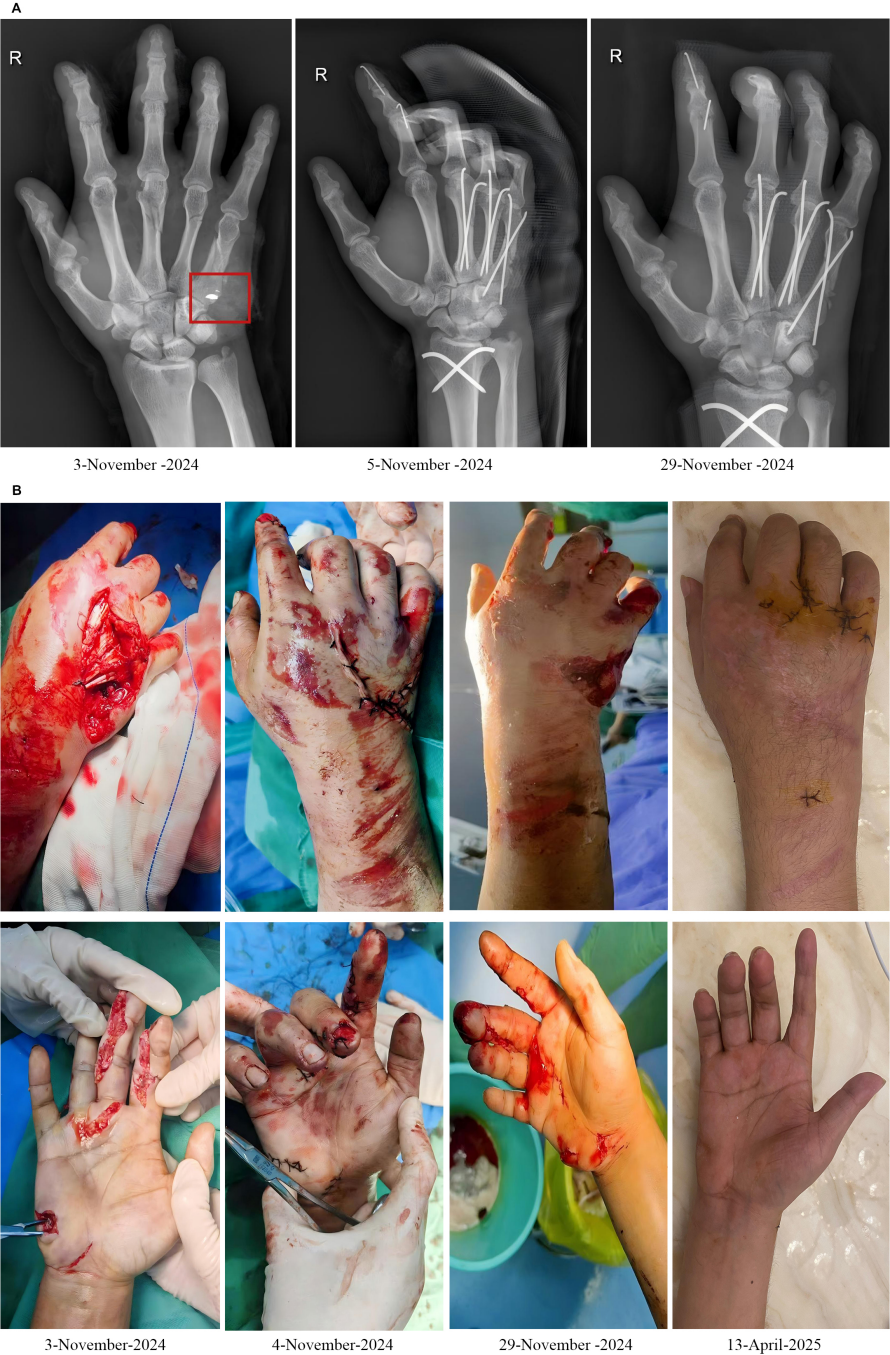
Radiographic images of the patient’s hand **(A)**. Photographs of the patient’s wound debridement, suturing, suture removal, and wound healing progress **(B)**.

After 15 days, the patient’s prognosis was favorable without significant discomfort. The physician confirmed that the right hand remained immobile in a plaster cast with dry and clean dressings. Despite restricted movement of the right index finger and slight limitations in the movement of the middle, ring, and little fingers and the wrist joint, the swelling had subsided significantly. The fingers appeared ruddy, with normal capillary refill and normal tension. The wound was dry without exudate and showed no obvious signs of infection. The patient was discharged. On day 161, following K-wire removal, microbial identification from a wound swab sample revealed *S. epidermidis*, a normal environmental flora, and the wound was healing well (**Figure 2**). The patient’s daily activities were not significantly impacted.

### Metal surface characterization and biofilm assays

Stainless steel sheets (0.5×10×10 mm) were classified into four types based on their surface characteristics, namely, smooth (SS316-P, TC4-P, and SS304-P with polished <0.01 µm) and rough (SS304-R). Three pieces of each sample were placed on a transparent 24-well polystyrene plate. Then, 1.5 ml of TSB culture medium containing 1.5×10^8^ CFU/ml of bacterial culture was added to each well to assess *in vitro* biofilm formation. Growth and sterile control groups were established. After incubation, the samples were washed three times with phosphate-buffered saline (PBS). For the *in vitro* biofilm formation assay, 1 ml of 0.5% trypsin was added to each of the cultures with incubation at 37°C for 5 minutes, followed by centrifugation at 3000 rpm for 7 minutes to remove the trypsin. The isolated bacterial pellets were serially diluted up to 10^4^ times, after which 10 µL of each diluted bacterial suspension was inoculated onto Columbia blood agar plates, and the number of colony-forming units (CFUs) was counted after 24 hours of incubation at 37°C (Ha et al., 2005; Paulitsch-Fuchs et al., 2022).

The biofilm-forming potential of the bacteria was quantified using the crystal violet microtiter plate assay, with modifications as described by Wcisłek et al (Wcisłek et al., 2025). Under identical culture conditions and after washing, *S. arlettae* and *S. aureus* biofilms on the surfaces of the materials were stained with crystal violet, and absorbances at 590 nm were measured using a microplate reader. With reference to the Stepanović classification, biofilm formation on metal surfaces was categorized as non-biofilm forming (OD ≤ ODc), weak (ODc < OD ≤ 2ODc), moderate (2ODc < OD ≤ 4ODc), or strong (OD > 4ODc), where ODc was defined as the mean OD of the negative control plus three standard deviations (Stepanovic et al., 2000). *S. aureus* ATCC 29213 was used as the positive control, and blank metal served as the negative control. All measurements were performed in triplicate.

**Figure 3:**
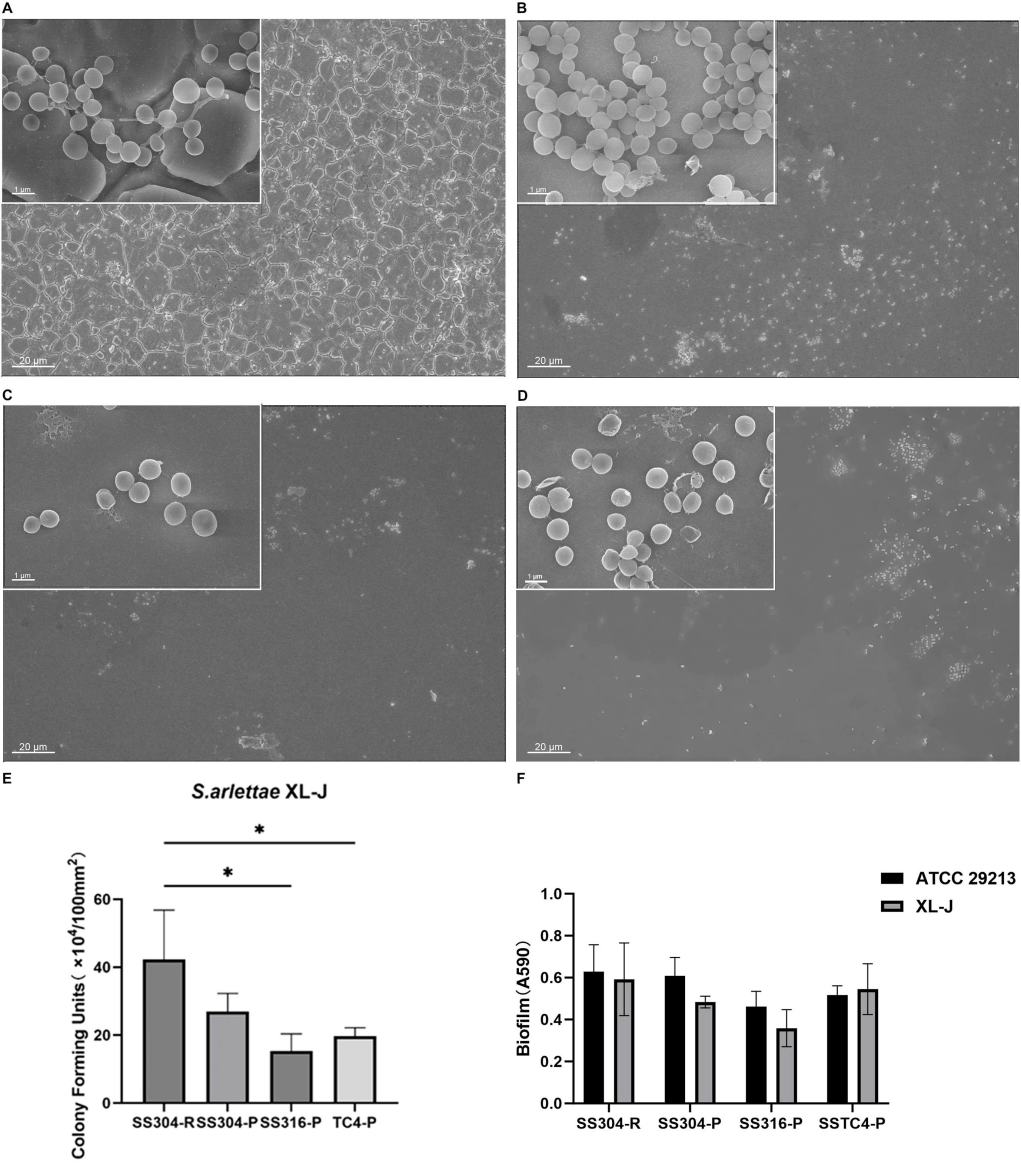
Scanning electron microscopy was used to evaluate bacterial adhesion to differently modified metal surfaces after immersion in an *S. arlettae* suspension for 24 hours. (×500, and ×10,000) Images of the biofilms on SS304-R **(A)**, SS304-P **(B)**, SS316-P **(C)**, and TC4-P **(D)**. The CFU counts of *S. arlettae* colonies on the four different metal fragments, namely, stainless steel (SS316-P), titanium alloy (TC4-P), stainless steel (SS304-P), and stainless steel (SS304-R), are presented as mean ± standard deviation SD and were analyzed by one-way ANOVA and Tukey’s multiple comparisons test. A *p-*value < 0.05 was considered statistically significant; an asterisk (*) indicates p < 0.05 **(E)**. Biofilm mass was quantified by measuring sample absorbance at 590 nm using a spectrophotometer. Data are expressed as mean ± SD and were analyzed using two-way ANOVA and Tukey multiple comparison test. A *p*-value < 0.05 was considered statistically significant **(F)**.

Finally, after the *in vitro* biofilm formation experiments, scanning electron microscopy (SEM) was used to evaluate the submicron-scale morphology (**Figure 3**).

## Results

The SEM results revealed that most *S. arlettae* cells aggregated into overlapping clusters on the SS304-R and SS304-P surfaces, showing a grape-like morphology. While the bacteria on the SS316-P and TC4-P surfaces tended to cluster, they did not overlap or cover one another, forming only a single layer with no grape-like appearance. Compared to bacteria grown on TC4-P, those on SS316-P were more separated, typically with only two or three bacteria together and no evidence of chain formation or overlap. Conversely, SEM images showed the presence of biofilm-like structures on the TC4-P surface, exhibiting a mushroom-like morphology with localized aggregation into clusters.

In terms of bacterial counts: *S. arlettae* on the SS316-P surface showed the lowest bacterial count of (1.53 ± 0.50) × 10^5^/100 mm², followed by the TC4-P surface with a value of (1.97 ± 0.25) × 10^5^/100 mm², the SS304-P surface with (2.70 ± 0.53) × 10^5^/100 mm², and the SS304-R surface with (4.23 ± 1.50) × 10^5^/100 mm². One-way ANOVA with Tukey’s multiple comparisons test indicated that SS304-R had significantly higher CFU counts than TC4-P and SS316-P (p < 0.05).

The results of the crystal violet staining indicated that *S. arlettae* formed the greatest amount of biofilm on SS304-R, with an OD value of 0.59 ± 0.17, which was higher than that on TC4-P (0.54 ± 0.12), SS304-P (0.48 ± 0.03), and SS316-P (0.36 ± 0.09).

The mean values for biofilm formation by *S. aureus* ATCC 29213 in each group were: SS304-R (0.63 ± 0.13), SS304-P (0.61 ± 0.09), SS316-P (0.46 ± 0.07), and TC4-P (0.52 ± 0.04). Two-way ANOVA showed a significant row factor (row factor: F (3, 16) = 3.959, *p* = 0.0275), indicating that biofilm formation differed significantly among the tested materials (*p* < 0.05). However, no significant differences between the two bacterial species were observed in pairwise comparisons.

Overall, the total mean value for biofilm formation by *S. aureus* ATCC 29213 was 0.55, while that for *S. arlettae* was 0.50, indicating that *S. aureus* ATCC 29213 produced a greater biofilm mass than *S. arlettae*; both strains exhibited a moderate biofilm production capacity.

## Discussion

Using methods such as swab culture (Levine technique), we isolated and identified *S. arlettae* from deep tissue samples collected from the same site during both debridement and the post-operative period (Kallstrom, 2014; Dudareva et al., 2021). *S. arlettae* does not form part of the normal human skin flora, excluding the possibility of contamination. Following 16S rRNA sequencing and phylogenetic tree construction (Neighbor-Joining Algorithm N-J), it was clearly observed that all *Staphylococcus* spp. clustered well together. Among them, the bootstrap values of *S.arlettae* and NR 024664.1 > 90, indicating it is reliable that they belong to the *S.arlettae* branch. This further confirms its pathogenic role as a pathogen, leading to the inference that the infection was caused by foreign material invading the wound (Metsemakers et al., 2018; Govaert et al., 2020). Interestingly, there was no growth of common *S. aureus* on the culture plates. Studies have shown that *S. arlettae* and other coagulase-negative staphylococci exhibit growth inhibition and quorum quenching against *S. aureus*, resulting in reduced colonization of *S. aureus* in the presence of *S. arlettae* isolates (Peng et al., 2019; Park et al., 2021).

The present patient experienced significant pain in the wound for a week after surgery. This was likely due to the inflammatory response caused by bacterial proliferation at the wound site, thereby prolonging wound healing (Gardner et al., 2001). A crucial virulence attribute of *S. arlettae* is its ability to form biofilms. Virulence factors produced by this species include various adhesion molecules, such as the laminin-binding protein (eno) and bone sialoprotein-binding protein (Bbp), as well as the gelatinase (Szafraniec et al., 2025). Bbp plays a central role in mediating bacterial adhesion to bone tissue, which is particularly critical in the pathogenesis of orthopedic implant-related infections (such as osteomyelitis and prosthetic joint infections) (Campoccia et al., 2009; Aamot et al., 2012). Laminin-binding proteins enable the binding of the bacteria to laminin, thereby facilitating the spread of infection to other tissues, while its polysaccharide components allow persistent attachment to foreign materials (Huebner and Goldmann, 1999; Carneiro et al., 2004). Becker et al. reported the detection of this bacterium in treatment-table water lines in hospitals and aerosols, on contact lenses and mobile phones, facilitated by bacterial genome-encoded genes/operons for resistance against copper, cobalt-zinc-cadmium, and mercury (Stanley et al., 2008; Dinakaran et al., 2012; Szymańska and Sitkowska, 2013; Kurli et al., 2018; Kalaiselvan et al., 2022).

Beyond the biological characteristics of the bacteria, this study found that their adhesion and proliferation on metal surfaces were also influenced by the type and physical properties of the material. Significantly lower CFU counts were observed on the SS316-P and TC4-P samples compared to SS304-R (*p* < 0.05). Among the stainless-steel materials, rough surfaces were associated with greater bacterial adhesion and proliferation, enabling a higher degree of colonization compared to smooth surfaces. Consequently, bacterial adhesion and biofilm formation were more active on SS304-R samples. The higher biocompatibility of TC4-P provided favorable surface conditions for biofilm formation, while the hydrophobicity of the SS316-P material, characterized by low surface free energy, adversely influenced the two-dimensional expansion of biofilms (Koseki et al., 2014). Consistent with these findings, the quantitative biofilm analysis further confirmed that biofilm formation was significantly influenced by the surface characteristics and material type (*p* < 0.05).

A Swiss study reported the identification of the blaARL gene, encoding a novel β-lactamase, from *S. arlettae*, and confirmed that the presence of this functional β-lactamase could compromise penicillin therapy (Andreis et al., 2017). An *S. arlettae* strain isolated in China was found to contain a novel multidrug-resistant plasmid, pSA-01, harboring nine antibiotic resistance genes, including cfr, erm(C), tet(L), erm(T), aadD, fosD, fexB, aacA-aphD, and erm(B). This was the first detection of co-localization of the cfr and fosD genes on a plasmid, potentially limiting antimicrobial treatment options (Liu et al., 2017). Therefore, wounds containing foreign bodies may appear minor and asymptomatic; however, if these foreign bodies are not detected during treatment, they can lead to serious sequelae days, months, or even years after the initial trauma (Goldstein and Imbriglia, 1986; Saaiq, 2014; Sherman and Murray, 2017).

In summary, due to the possibility of fracture-related infection (FRI) bacteremia, perioperative antibiotic prophylaxis, along with accurate pathogen identification and rapid testing of antimicrobial susceptibility, are necessary for the correct and effective implementation of treatment (Lack et al., 2015; Depypere et al., 2020; Obremskey et al., 2020). However, due to the adhesive properties of this staphylococcus species on metals, immediate closure of the wound after debridement may lead to disastrous outcomes (Karley et al., 2019). These multidrug-resistant strains can invade adjacent tissues, accelerating clinical deterioration, especially in immunocompromised hosts (Societies W U o W H, 2008; World Health Organization, 2017). Therefore, the use of multiple debridement and dressing changes with delayed wound closure, the utilization of antibiotic-coated metal implants for surgical fixation, and the application of moist wound healing are considered preferable (Metsemakers et al., 2017; O’Toole et al., 2021).

Our study has several limitations. First, as *S. arlettae* is a rare pathogen in human infections, its occurrence and transmission require further monitoring given the small sample size of the present study. Second, the study did not specifically investigate the expression levels of genes regulating biofilm formation and antibiotic resistance.

## Summary

*S. arlettae* can colonize medical devices and the surrounding environment with low bacterial loads. Although host immunity can typically control such colonization, it might be clinically misjudged as contamination, and thus not treated sufficiently. It is noteworthy that following procedures such as dental surgery and total hip replacement, where materials are implanted into deep tissues, *S. arlettae* can invade exposed wounds or disseminate hematogenously via orthopedic instruments or prosthetic joints. The presence of implants can significantly enhance the rate of bacterial proliferation, their biofilm-forming capacity, and drug resistance, thereby increasing the risk of postoperative infection. When laboratories report the presence of *S. arlettae*, it is advisable to include a comment on its clinical significance and communicate with clinicians regarding the potential need for repeat sampling or further molecular or mass spectrometric identification.

## Data Availability

The datasets presented in this study can be found in online repositories. The names of the repository/repositories and accession number(s) can be found below: https://www.ncbi.nlm.nih.gov/genbank/, PX472910.1.
