# Supplementary material for: Wound infection caused by Staphylococcus arlettae: a case report and metal characterization
Source: Front Cell Infect Microbiol. 2026 Mar 5;16:1780396. doi: 10.3389/fcimb.2026.1780396 (PMC12999913; doi:10.3389/fcimb.2026.1780396)
Supplement: Supplementary file 1 [file Table1.docx]

| **Table S1**.Microbiological sampling timeline and specimen characteristics in the present case | | | | | |
| --- | --- | --- | --- | --- | --- |
| **Sampling time point** | **Clinical context** | **Anatomical site** | **Specimen site** | **Specimen type** | **Microbiological result** |
| Hospital day 1 (Initial surgery) | Wound debridement | Proximal ulnar–volar aspect of the right wrist | Deep soft tissue extending to bone | Wound swab (swab culture) | *Staphylococcus arlettae* isolated |
| Hospital day 5 (Postoperative) | Delayed wound healing | Proximal ulnar–volar aspect of the right wrist | The same deep wound postoperatively | Wound swab (swab culture) | *Staphylococcus arlettae* isolated |
| Day 161 (Following K-wire removal) | Post–implant removal assessment | Proximal ulnar–volar aspect of the right wrist | Wound bed | Wound swab (swab culture) | *Staphylococcus epidermidis* isolated |
